# Induction of secondary metabolism of *Aspergillus terreus* ATCC 20542 in the batch bioreactor cultures

**DOI:** 10.1007/s00253-015-7157-1

**Published:** 2015-11-25

**Authors:** Tomasz Boruta, Marcin Bizukojc

**Affiliations:** Faculty of Process and Environmental Engineering, Department of Bioprocess Engineering, Lodz University of Technology, ul. Wolczanska 213, 90-924 Lodz, Poland

**Keywords:** *Aspergillus terreus*, Secondary metabolites, Lovastatin, (+)-Geodin

## Abstract

Cultivation of *Aspergillus terreus* ATCC 20542 in a stirred tank bioreactor was performed to induce the biosynthesis of secondary metabolites and provide the bioprocess-related insights into the metabolic capabilities of the investigated strain. The activation of biosynthetic routes was attempted by the diversification of process conditions and growth media. Several strategies were tested, including the addition of rapeseed oil or inulin, changing the concentration of nitrogen source, reduction of chlorine supply, cultivation under saline conditions, and using various aeration schemes. Fifteen secondary metabolites were identified in the course of the study by using ultra-high performance liquid chromatography coupled with mass spectrometry, namely mevinolinic acid, 4a,5-dihydromevinolinic acid, 3α-hydroxy-3,5-dihydromonacolin L acid, terrein, aspulvinone E, dihydroisoflavipucine, (+)-geodin, (+)-bisdechlorogeodin, (+)-erdin, asterric acid, butyrolactone I, desmethylsulochrin, questin, sulochrin, and demethylasterric acid. The study also presents the collection of mass spectra that can serve as a resource for future experiments. The growth in a salt-rich environment turned out to be strongly inhibitory for secondary metabolism and the formation of dense and compact pellets was observed. Generally, the addition of inulin, reducing the oxygen supply, and increasing the content of nitrogen source did not enhance the production of examined molecules. The most successful strategy involved the addition of rapeseed oil to the chlorine-deficient medium. Under these conditions, the highest levels of butyrolactone I, asterric acid, and mevinolinic acid were achieved and the presence of desmethylsulochrin and (+)-bisdechlorogeodin was detected in the broth. The constant and relatively high aeration rate in the idiophase was shown to be beneficial for terrein and (+)-geodin biosynthesis.

**Electronic supplementary material:**

The online version of this article (doi:10.1007/s00253-015-7157-1) contains supplementary material, which is available to authorized users.

## Introduction

Fungi are well-known for their ability to biosynthesize a vast array of low molecular weight molecules with sophisticated structures and various biological functions. These compounds, traditionally referred to as secondary metabolites, are not necessary for the operation of energetic and growth systems of the cell, yet their formation has been established in the course of evolution as being advantageous for the producing organism (Demain and Fang 2000). The examples of fungal secondary metabolites are polyketides, non-ribosomal peptides, and terpenes (Keller et al. 2005). Importantly, the secondary metabolic machineries of fungi are considered to be rich sources of potential drug candidates and other useful substances. The widely recognized examples of industrially and medically relevant metabolites of fungal origin are penicillin, a beta-lactam antibiotic produced by *Penicillium chrysogenum*, and lovastatin, a cholesterol-lowering drug secreted by *Aspergillus terreus* (Alberts et al. 1980; Brakhage 1998; Tobert 2003).

The cascades of cellular events leading to the biosynthesis of secondary metabolites are controlled at multiple levels (Brakhage 2013). As opposed to primary metabolic pathways, the activity of secondary pathways is typically observed only under specific conditions. The evolutionarily shaped set of environmental factors and complex molecular mechanisms associated with the activation of biosynthetic genes is generally very difficult or even impossible to fully elucidate. The genes may remain silent under the applied cultivation strategy due to a lack of required stimuli and, as a result, the corresponding molecules are not produced. To address this issue, the methods of genome mining have been devised to trigger the formation of the respective secondary metabolites by using the tools of genetic engineering (Bok et al. 2015; Bergmann et al. 2007; Brakhage and Schroeckh 2011; Guo and Wang 2014; Ochi and Hosaka 2013). An alternative strategy involves the examination of diverse cultivation conditions and media compositions in order to uncover the metabolic repertoire of strains under investigation (Bode et al. 2002). The rationale behind this bioprocess-oriented approach is that the resemblance of natural stimuli leading to biosynthesis of secondary metabolites can, in principle, be achieved through extensive testing of various growth conditions in the laboratory setting. The typically considered process-related variables include carbon and nitrogen sources, pH, aeration, temperature, and the concentration of nutrients. The objective of the underlying procedures is to mimic the environmental conditions and signals that elicit the molecular response associated with awakening of the corresponding genes. The regulation of fungal secondary metabolism was shown to be integrated with stress response mechanisms (Duran et al. 2010; Roze et al. 2011; Yin et al. 2013). Therefore, subjecting the target strain to oxidative, osmotic, and other forms of stress may reveal the previously unknown part of the metabolome. Not only do such experiments open the door for the exploration of fungal metabolic landscapes but they also allow for the identification of key process-related factors that stimulate biosynthetic pathways. Moreover, they provide the preliminary data to facilitate further bioprocess development and optimizations efforts oriented towards reaching high titers, productivities, and yields of the desired molecules. By investigating the metabolic profiles under diverse conditions, one may not only search for the conditions that maximize the synthesis of the target compounds but, simultaneously, investigate the factors that reduce or eliminate the formation of unwanted by-products. Monitoring of the broad spectrum of metabolites during the cultivation process leads to the more complete picture of the biosynthetic capabilities of the employed strains (Sarkar et al. 2012).

*Aspergillus terreus* is a textbook example of an industrially exploited filamentous fungus. Its major metabolite lovastatin, also known as mevinolin, is applied in cholesterol lowering treatments. This species has been a subject of many experimental works devoted to the natural products and the results clearly indicate that its biosynthetic potential reaches far beyond the formation of lovastatin. The search in the *Antibase 2014* database of secondary metabolites with the query “*Aspergillus terreus*” yields 165 chemical structures of compounds that have been reported for this fungus throughout the years of research. Importantly, this relatively high number corresponds to the overall number of investigated strains and isolates, encompassing the ones isolated from soil, marine environments, and plant material. Knowing the long-established industrial status of *A. terreus*, it is justified to examine the true metabolic capabilities of a single, biotechnologically relevant strain and monitor its chemical catalogue in the context of the diversified growth media and cultivation conditions. A well-suited candidate for this purpose is *A. terreus* ATCC 20542, a wild-type parent strain of industrial lovastatin-overproducing mutants (Alberts et al. 1980; Monaghan et al. 1980). Its secondary metabolic repertoire has been a subject of previous qualitative investigations conducted for the cultures propagated on the solid media (Samson et al. 2011) and in shake flasks (Boruta and Bizukojc 2014). Vinci et al. (1991) described a number of metabolic by-products detected during the industrial-scale production of lovastatin, including asterric acid and sulochrin; however, the study was conducted with the use of mutant strains derived from *A. terreus* ATCC 20542 and it was focused on the reduction of co-metabolites formation. The comprehensive bioreactor-based analysis of secondary metabolic spectrum of the wild-type parent strain has not been reported previously.

This paper addresses the media- and conditions-dependent induction of secondary metabolites biosynthesis by *A. terreus* ATCC 20542 in a 5-l stirred tank bioreactor. The presented qualitative and quantitative analysis considered the bioprocess-related factors associated with the induction of biosynthetic pathways in *A. terreus* that can be further tested for other fungal systems. The mini-database of mass spectra and fragmentation patterns of encountered molecules is also provided, the resource that may be used in the future analytical studies on *A. terreus* metabolism.

## Materials and methods

### Strain

*Aspergillus terreus* ATCC 20542 was used throughout the study.

### Media and cultivation conditions

The composition of the solid agar medium used for the production of conidia was as follows: malt extract (20 g l^−1^), casein peptone (5 g l^−1^), agar (30 g l^−1^).

The growth media contained the following: lactose, 20 g l^−1^ (10 g l^−1^ in the preculture); KH_2_PO_4_, 1.51 g l^−1^; yeast extract (BD, USA), 2 g l^−1^ in all bioreactor runs except R3 and R4, 4 g l^−1^ in run R3, 0.5 g l^−1^ in run R4, 8 g l^−1^ in the preculture; MgSO_4_·7H_2_O, 0.52 g l^−1^; ZnSO_4_·7H_2_O, 1 mg l^−1^; Fe(NO)_3_·9H_2_O, 2 mg l^−1^; biotin, 0.04 mg l^−1^. One milliliter of the following solution was added per liter of medium: H_3_BO_3_, 65 mg l^−1^; MnSO_4_·1H_2_O, 43 mg l^−1^; CuSO_4_·5H_2_O, 250 mg l^−1^; Na_2_MoO_4_·2H_2_O, 50 mg l^−1^. Inulin from chicory (Sigma-Aldrich, USA) at the concentration of 5 g l^−1^ served as an additional carbon source in the run R2. With the exception of the runs R1, R7 and the preculture for the run R7, the media contained NaCl at the concentration of 0.4 g l^−1^. In the run R1, the concentration of NaCl was equal to 150 g l^−1^. In the run R7, and the corresponding preculture Na_2_SO_4_ (0.486 g l^−1^) was used as a source of sodium instead of NaCl.

In the runs R1, R2, R3, and R4, the silicone oil Antifoam A (Sigma-Aldrich, USA) was employed as a foam suppresser, while in the runs R5, R6, R7, and R8 cold-filtered rapeseed oil (ZT Kruszwica SA, Poland) was used (10 ml per liter of medium) as an anti-foam agent and an additional source of carbon. Five milliliters of rapeseed oil per liter of medium was added prior to inoculation and the remaining volume was added during the initial 24 h of cultivation.

The pH value of culture media was adjusted to 6.5 by using NaOH. The media in the bioreactor were autoclaved at 121 °C for 90 min.

Induction of sporulation was achieved by propagating the fungus for 10 days on malt agar slants. The conidia were transferred to shake flasks by washing with the medium to reach approximately 10^9^ conidia per liter of the preculture. Three hundred milliliters of the preculture was prepared by cultivating the fungus for 24 h in two shake flasks (total volume 500 ml, working volume 150 ml). The rotary shaker speed was set to the constant value of 110 rpm and the temperature was maintained at 30 °C. The inoculation with the 24-h preculture resulted in the initial biomass concentration in the bioreactor between 0.1 and 0.15 g l^−1^.

Experimental runs were performed for 168 h at 30 °C in a stirred tank bioreactor BIOSTAT® B Plus (Sartorius, Germany) with the working volume of 5.4 l. In all runs except R5 and R8, the initial value of *vvm* (ratio of air flow rate and bioreactor volume) was set to 0.28 l_air_ l^−1^ min^−1^, the initial rotary speed of the impeller was equal to 200 rpm (rotations per minute), and the dissolved oxygen saturation level was controlled at the level of 20 % by adjusting the air flow rate and impeller speed. In the run R5, the oxygen saturation level was not controlled, the agitation speed was maintained at 200 rpm, and the *vvm* value was kept at 0.28 l_air_ l^−1^ min^−1^ except the period between 32 and 47 h, when the intentional shutdown of aeration took place and the *vvm* value was equal to 0. In the run R8, there was no control of oxygen saturation level, the impeller speed was constant at 240 rpm, and the *vvm* was constant at 0.75 l_air_ l^−1^ min^−1^.

### Analytical methods

Secondary metabolites were analyzed by using the ultra-high performance liquid chromatography (UPLC® Acquity)-mass spectrometry (SYNAPT G2) system (Waters, USA). The chromatographic procedure was executed as previously described (Bizukojc et al. 2012). The mass spectrometry analysis was conducted both in positive and negative electrospray ionization modes. The following parameters were applied: the temperature of the source was set to 120 °C; desolvation temperature was 200 °C in ESI+ mode and 400 °C in ESI− mode; voltage was 3 kV for the capillary, 40 V for the sampling cone, and 4 V for the extraction cone; flow rate of desolvation gas (nitrogen) was equal to 500 l h^−1^ in ESI+ and 1000 l h^−1^ in ESI− mode. The absorbance spectra generated by using the photodiode array (PDA) detector were scrutinized in concert with the chromatograms and mass spectra in the process of metabolite detection and identification. The database of natural products *AntiBase 2014*: *The Natural Compound Identifier* (Laatsch 2014) was consulted in the course of the qualitative analysis of molecules.

Quantitative analyses of mevinolinic acid, (+)-geodin, and terrein were carried out by means of UPLC® Acquity (Waters, USA) with the photodiode array detector at λ = 238 nm (for mevinolinic acid) and λ = 280 nm (for (+)-geodin and terrein). Asterric acid and butyrolactone I were assayed by the LC-MS/MS method implemented in TargetLynx™ (Waters, USA) in a positive and negative electrospray ionization mode, respectively. The quantification traces for asterric acid and butyrolactone I were defined as *m*/*z* = 331.086 and *m*/*z* = 423.146, respectively. The calibration curves for the quantitative assays were developed by using standard solutions prepared from commercially available metabolites. The standard solution of mevinolinic acid was formulated by using the method presented in earlier study Casas López et al. (2003). The standard solution of (+)-geodin was prepared by dissolving (+)-geodin (Santa Cruz Biotechnology, USA) in a solution of acetonitrile and water (1:1 *v*/*v*). The standard solutions of asterric acid (Enzo Life Sciences, USA) and butyrolactone I (Enzo Life Sciences, USA) were prepared in acetonitrile, while terrein (Sigma-Aldrich, USA) was dissolved in water. The reference samples of questin and sulochrin were kindly provided by Prof. Dr. Isao Fujii, Iwate Medical University, Japan.

Image analysis regarding fungal morphology was performed as previously described (Gonciarz and Bizukojc 2014).

### Molecular formulae of secondary metabolites, *m*/*z* of pseudomolecular ion peaks and experimental errors

(+)-Bisdechlorogeodin (C_17_H_14_O_7_; calculated *m*/*z* [M + H]^+^ = 331.0812; experimental *m*/*z* [M + H]^+^ = 331.0857; error Δ*m*/*z* = +0.0045); (+)-erdin (C_16_H_10_Cl_2_O_7_; calc. *m*/*z* [M + H]^+^ = 384.9876; exper. *m*/*z* [M + H]^+^ = 384.9872; Δ*m*/*z* = −0.0004); (+)-geodin (C_17_H_12_Cl_2_O_7_; calc. *m*/*z* [M + H]^+^ = 399.0033; exper. *m*/*z* [M + H]^+^ = 399.0057; Δ*m*/*z* = +0.0024); 3α-hydroxy-3,5-dihydromonacolin L acid (C_19_H_32_O_5_; calc. *m*/*z* [M-H]^−^ = 339.2177; exper. *m*/*z* [M-H]^−^ = 339.2166; Δ*m*/*z* = −0.0011); aspulvinone E (C_17_H_12_O_5_; calc. *m*/*z* [M-H]^−^ = 295.0612; exper. *m*/*z* [M-H]^−^ = 295.0606; Δ*m*/*z* = −0.0006); asterric acid (C_17_H_16_O_8_; calc. *m*/*z* [M + H]^+^ = 349.0918; exper. *m*/*z* [M + H]^+^ = 349.0882; Δ*m*/*z* = −0.0036); butyrolactone I (C_24_H_24_O_7_; calc. *m*/*z* [M-H]^−^ = 423.1449; exper. *m*/*z* [M-H]^−^ = 423.1462; Δ*m*/*z* = +0.0013); demethylasterric acid (C_16_H_14_O_8_; calc. *m*/*z* [M + H]^+^ = 335.0761; exper. *m*/*z* [M + H]^+^ = 335.0797; Δ*m*/*z* = +0.0036); desmethylsulochrin (C_16_H_14_O_7_; calc. *m*/*z* [M + H]^+^ = 319.0812; exper. *m*/*z* [M + H]^+^ = 319.0835; Δ*m*/*z* = +0.0023); dihydroisoflavipucine (C_12_H_17_NO_4_; calc. *m*/*z* [M + H]^+^ = 240.1230; exper. *m*/*z* [M + H]^+^ = 240.1268; Δ*m*/*z* = +0.0038); 4a,5-dihydromevinolinic acid (C_24_H_40_O_6_; calc. *m*/*z* [M + H]^+^ = 425.2898; exper. *m*/*z* [M + H]^+^ = 425.2870; Δ*m*/*z* = −0.0028); mevinolinic acid (C_24_H_38_O_6_; calc. *m*/*z* [M + H]^+^ = 423.2741; exper. *m*/*z* [M + H]^+^ = 423.2716; Δ*m*/*z* = −0.0025); questin (C_16_H_12_O_5_; calc. *m*/*z* [M + H]^+^ = 285.0758; exper. *m*/*z* [M + H]^+^ = 285.0770; Δ*m*/*z* = +0.0012); sulochrin (C_17_H_16_O_7_; calc. *m*/*z* [M + H]^+^ = 333.0969; exper. *m*/*z* [M + H]^+^ = 333.0997; Δ*m*/*z* = +0.0028); terrein (C_8_H_10_O_3_; calc. *m*/*z* [M + H]^+^ = 155.0703; exper. *m*/*z* [M + H]^+^ = 155.0705; Δ*m*/*z* = +0.0002)

## Results

**Fig. 1:**
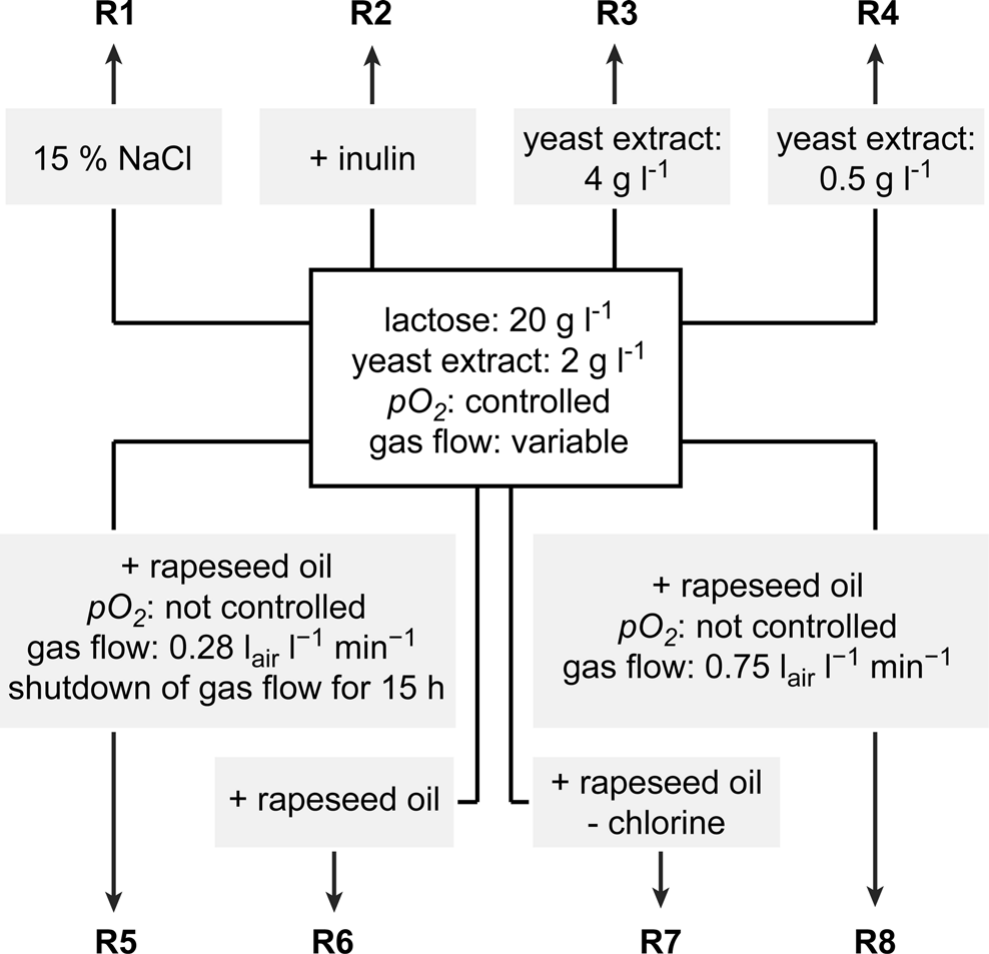
Scheme of experimental runs. Each run involved a number of modifications with respect to the set of conditions presented in the box. Run R1 involved the highly saline environment of 15 % (*w*/*v*) sodium chloride; inulin was added to the R2 medium; two different concentration values of yeast extract were tested in R3 and R4; rapeseed oil was supplemented in the runs R5, R6, R7, and R8; distinctive aeration strategies were applied in the runs R5 and R8; the reduction of chlorine content in the R7 run was achieved by eliminating sodium chloride from the medium

Three general approaches, employed separately or simultaneously, were tested for their capability to elicit biosynthesis of secondary metabolites in the bioreactor cultures of *A. terreus* ATCC 20542. The first strategy was to activate cellular stress response that might lead to the stimulation of biosynthetic metabolic routes. This was attempted by establishing high salt conditions, performing temporal shutdown of air flow in the bioreactor, using various aeration strategies, and minimalizing the level of chlorine in the medium. The second method involved the supplementation with an additional carbon source, namely inulin or rapeseed oil. The third approach relied on changing the concentration of nitrogen source in the medium. The scheme of the performed experimental runs is presented in Fig. 1 to illustrate the differences between the investigated cultures.

### Identification of secondary metabolites

Altogether, 15 secondary metabolites were identified in the cultivation broths. The metabolites were analyzed by the combined approach involving three methods. The first step was to analyze the mass and fragmentation of molecules by mass spectrometry, which led to the determination of the accurate mass values of metabolites and their fragments. The second step was based on retention time analysis. The third step was based on absorbance measurements at different wavelengths and analysis of UV spectra with the use of the photodiode array detector (PDA) for all metabolites. The results at each step where compared with the data obtained for standards or, whenever they were not available, with literature data. The metabolites detected in the study were all reported previously but their simultaneous production was never a subject of a comprehensive study involving bioreactor cultivation. The presence of mevinolinic acid, (+)-geodin, terrein, asterric acid, butyrolactone I, questin, and sulochrin was confirmed by considering the mass spectra, elution times, and absorbance data obtained for the available standard solutions. Recognition of 4a,5-dihydromevinolinic acid, 3α-hydroxy-3,5-dihydromonacolin L acid, (+)-erdin, (+)-bisdechlorogeodin, demethylasterric acid, aspulvinone E, dihydroisoflavipucine, and desmethylsulochrin was based on the thorough analysis of spectral data and its comparison with the available literature records. The observed fragmentation patterns of the detected metabolites with the suggested masses are presented in Table 1, while the collection of mass spectra is provided in the Supplementary material (Figs. S1–S15). For the majority of examined molecules, a strong and distinctive signal was observed in both positive and negative ionization modes. However, for a limited number of metabolites, the signals of significant intensity were found only in one of the employed ionization modes (Table 1). Under the applied analytical conditions, the fragmentation of mevinolinic acid, 4a,5-dihydromevinolinic acid, 3α-hydroxy-3,5-dihydromonacolin L acid, and (+)-geodin could be observed solely when the molecules were subjected to the positive ionization.

**Table 1:** Fragmentation patterns observed in mass spectra of secondary metabolites detected in bioreactor cultures of *A. terreus* ATCC 20542

Name of metabolite	Experimental *m*/*z* values of identified pseudo-molecular ions and fragmentation ions with the corresponding losses	
	ESI+ mode	ESI− mode
(+)-Bisdechlorogeodin	331.0857, [M + H]^+^; 299.0583, [M-CH_3_OH + H]^+^; 287.0940, [M-CO_2_ + H]^+^	329.0677, [M-H]^−^; 245.0809, [M-3CO-H]^−^; 167.0350, [M-C_9_H_6_O_3_-H]^−^
(+)-Erdin	384.9872, [M + H]^+^; 340.9987, [M-CO_2_ + H]^+^; 325.9790, [M + H-C_2_H_3_O_2_]^+^	382.9689, [M-H]^−^; 338.9831, [M-CO_2_-H]^−^
(+)-Geodin	399.0057, [M + H]^+^; 366.9813, [M-CH_3_OH + H]^+^; 355.0138, [M-CO_2_ + H]^+^; 339.9911, [M + H-COOCH_3_]^+^	396.9890, [M-H]^−^
3α-Hydroxy-3,5-dihydromonacolin L acid	323.2245, [M-H_2_O + H]^+^; 305.2083, [M-2H_2_O + H]^+^; 287.1976, [M-3H_2_O + H]^+^; 681.4574, [2M + H]^+^; 645.4343, [2M-2H_2_O + H]^+^; 627.4283, [2M-3H_2_O + H]^+^	339.2166, [M-H]^−^
Aspulvinone E	–	295.0606, [M-H]^−^
Asterric acid	349.0882, [M + H]^+^; 331.0857, [M-H_2_O + H]^+^; 299.0583, [M-H_2_O-CH_3_OH + H]^+^; 287.0940, [M-H_2_O-CO_2_ + H]^+^	347.0801, [M-H]^−^; 303.0897, [M-CO_2_-H]^−^; 271.0583, [M-CO_2_-CH_3_OH-H]^−^
Butyrolactone I	–	423.1462, [M-H]^−^; 379.1541, [M-CO_2_-H]^−^; 364.1277, [M-COOCH_3_-H]^−^
Demethylasterric acid	335.0797, [M + H]^+^; 317.0658, [M-H_2_O + H]^+^; 299.0583, [M-2H_2_O + H]^+^; 273.0737, [M-H_2_O-CO_2_ + H]^+^	333.0609, [M-H]^−^; 289.0686, [M-CO_2_-H]^−^; 245.0809, [M-2CO_2_-H]^−^; 167.0350, [M-C_8_H_6_O_4_-H]^−^
Desmethylsulochrin	319.0835, [M + H]^+^; 301.0710, [M-H_2_O + H]^+^; 195.0269, [M-C_7_H_8_O_2_ + H]^+^	317.0691, [M-H]^−^; 285.0392, [M-CH_3_OH-H]^−^; 241.0459, [M-CH_3_OH-CO_2_-H]^−^; 193.0154, [M-C_7_H_8_O_2_-H]^−^
Dihydroisoflavipucine	240.1268, [M + H]^+^; 142.0508, [M-C_6_H_10_O + H]^+^, 479.2446, [2M + H]^+^	-
4a,5-Dihydromevinolinic acid	425.2870, [M + H]^+^; 407.2763, [M-H_2_O + H]^+^; 305.2159, [M-H_2_O-C_5_H_10_O_2_ + H]^+^; 287.2050, [M-2H_2_O-C_5_H_10_O_2_ + H]^+^; 269.1897, [M-3H_2_O-C_5_H_10_O_2_ + H]^+^; 245.1892, [M-H_2_O-C_5_H_10_O_2_-CH_3_COOH + H]^+^; 227.1812, [M-2H_2_O-C_5_H_10_O_2_-CH_3_COOH + H]^+^; 201.1625, [M-H_2_O-C_5_H_10_O_2_-CH_3_COOH-C_2_H_4_O + H]^+^; 203.1810, [M-H_2_O-C_5_H_10_O_2_-C_4_H_6_O_3_ + H]^+^	423.2722, [M-H]^−^
Mevinolinic acid	423.2716, [M + H]^+^; 405.2600, [M-H_2_O + H]^+^; 303.1969, [M-H_2_O-C_5_H_10_O_2_ + H]^+^; 285.1876, [M-2H_2_O-C_5_H_10_O_2_ + H]^+^; 267.1725, [M-3H_2_O-C_5_H_10_O_2_ + H]^+^; 243.1756, [M-H_2_O-C_5_H_10_O_2_-CH_3_COOH + H]^+^; 225.1644, [M-2H_2_O-C_5_H_10_O_2_-CH_3_COOH + H]^+^; 199.1478, [M-H_2_O-C_5_H_10_O_2_-CH_3_COOH-C_2_H_4_O + H]^+^; 201.1625, [M-H_2_O-C_5_H_10_O_2_-C_4_H_6_O_3_ + H]^+^	421.2598, [M-H]^−^
Questin	285.0770, [M + H]^+^	283.0572, [M-H]^−^
Sulochrin	333.0997, [M + H]^+^; 301.0710, [M-CH_3_OH + H]^+^; 209.0444, [M-C_7_H_8_O_2_ + H]^+^	331.0851, [M-H]^−^; 299.0541, [M-CH_3_OH-H]^−^
Terrein	155.0705, [M + H]^+^; 137.0610, [M-H_2_O + H]^+^; 109.0642, [M-H_2_O-CO + H]^+^	-

**Fig. 2:**
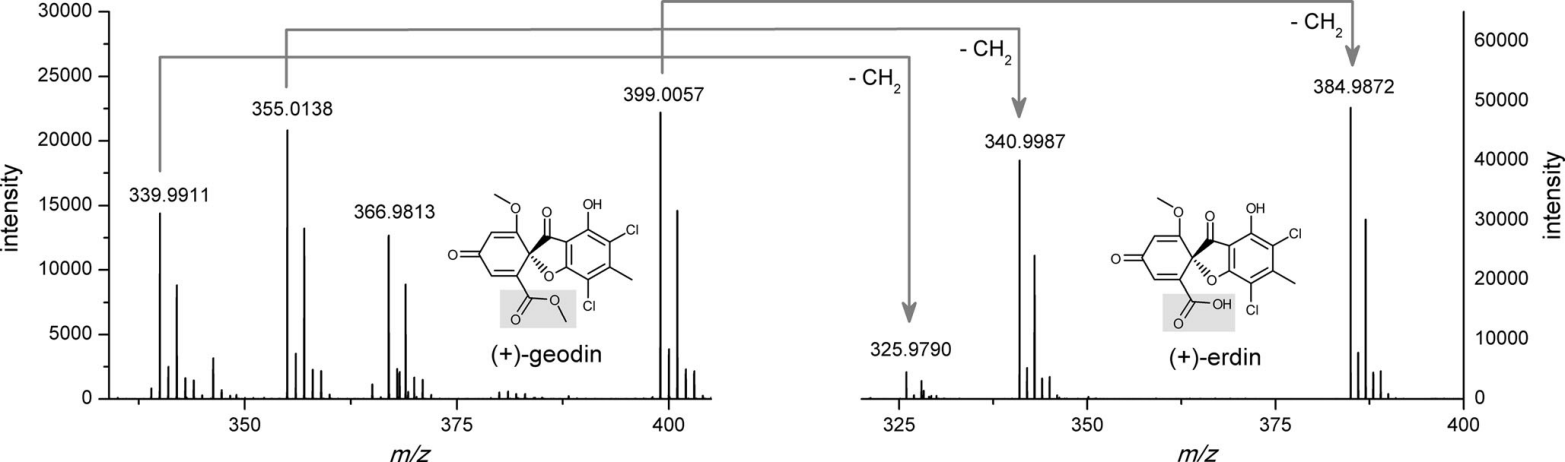
Scheme of fragmentation pattern observed in mass spectra of (+)-geodin and (+)-erdin. The (+)-geodin peaks at *m*/*z* = 339.9911, 355.0138, and 399.0057 differ by the Δ*m*/*z* value corresponding to the mass of “CH_2_” (theoretical Δ*m*/*z* = 14.0157) from the corresponding (+)-erdin peaks at *m*/*z* = 325.9790, 340.9987, and 384.9872, respectively. The relatively small deviations from the theoretical Δ*m*/*z* values are due to the experimental error

The examined mass spectra revealed a fragmentation pattern shared between (+)-erdin and (+)-geodin, which in fact is a methyl ester of (+)-erdin (Fig. 2). The (+)-geodin peaks at *m*/*z* = 339.9911, 355.0138, and 399.0057 differed by the Δ*m*/*z* value corresponding to the mass of “CH_2_” from the corresponding (+)-erdin peaks at *m*/*z* = 325.9790, 340.9987, and 384.9872, respectively. The theoretical *m*/*z* value of “CH_2_” group is equal to 14.0157, which fits well in the presented pattern, if the experimental error is considered. This difference in *m*/*z* reflected the structural relationship between the two molecules (Fig. 2). The analogous observation was made for two other pairs of detected metabolites. The peaks of asterric acid at *m*/*z* = 349.0882, 331.0857, and 287.0940 had their counterparts in the demethylasterric acid spectrum at *m*/*z* = 335.0797, 317.0658, and 273.0737, respectively (Table 1). Finally, the sulochrin peaks at *m*/*z* = 333.0997 and 209.0444 had their corresponding “-CH_2_” peaks in the spectrum of desmethylsulochrin (also referred to as demethylsulochrin) at *m*/*z* = 319.0835 and 195.0269, respectively (Table 1).

Another fragmentation pattern was observed to be shared by mevinolinic acid, which is a β-hydroxy acid form of mevinolin, and 4a,5-dihydromevinolinic acid, a β-hydroxy acid form of 4a,5-dihydromevinolin (Albers-Schönberg et al. 1981). All the mass peaks of 4a,5-dihydromevinolinic acid had their “-2H” counterparts (theoretical Δ*m*/*z* = 2.01565) in the mevinolinic acid spectrum (Table 1). Such behavior was expected, since these two molecules differ structurally only by two hydrogen atoms (Albers-Schönberg et al. 1981).

The mass spectrum of (+)-bisdechlorogeodin including the peaks at *m*/*z* = 331.0857, 299.0583, and 287.0940 is in agreement with the positive-ionization data presented by Nielsen and Smedsgaard (2003). The fragment peak at *m*/*z* = 142.0508 observed for dihydroisoflavipucine (Table 1) corresponded well with the peak reported previously at *m*/*z* = 142.1 by Zhou (2012). For aspulvinone E, a yellow compound previously isolated from *A. terreus* (Seto 1979), the relatively strong absorbance was observed at 400 nm. It agrees well with literature data, since yellow substances absorb light at 400 nm (Pavia et al. 2015). The UV absorption maxima of mevinolinic acid, 4a,5-dihydromevinolinic acid, questin, asterric acid, demethylasterric acid, sulochrin, (+)-bisdechlorogeodin, terrein, butyrolactone I, dihydroisoflavipucine, (+)-erdin, and (+)-geodin were in agreement with the previously published data (Albers-Schönberg et al. 1981; Calam et al. 1947; Curtis et al. 1960; Guo et al. 2012; Nielsen and Smedsgaard 2003; Zhou 2012).

### Influence of growth media and cultivation conditions on biosynthesis of secondary metabolites

**Fig. 3:**
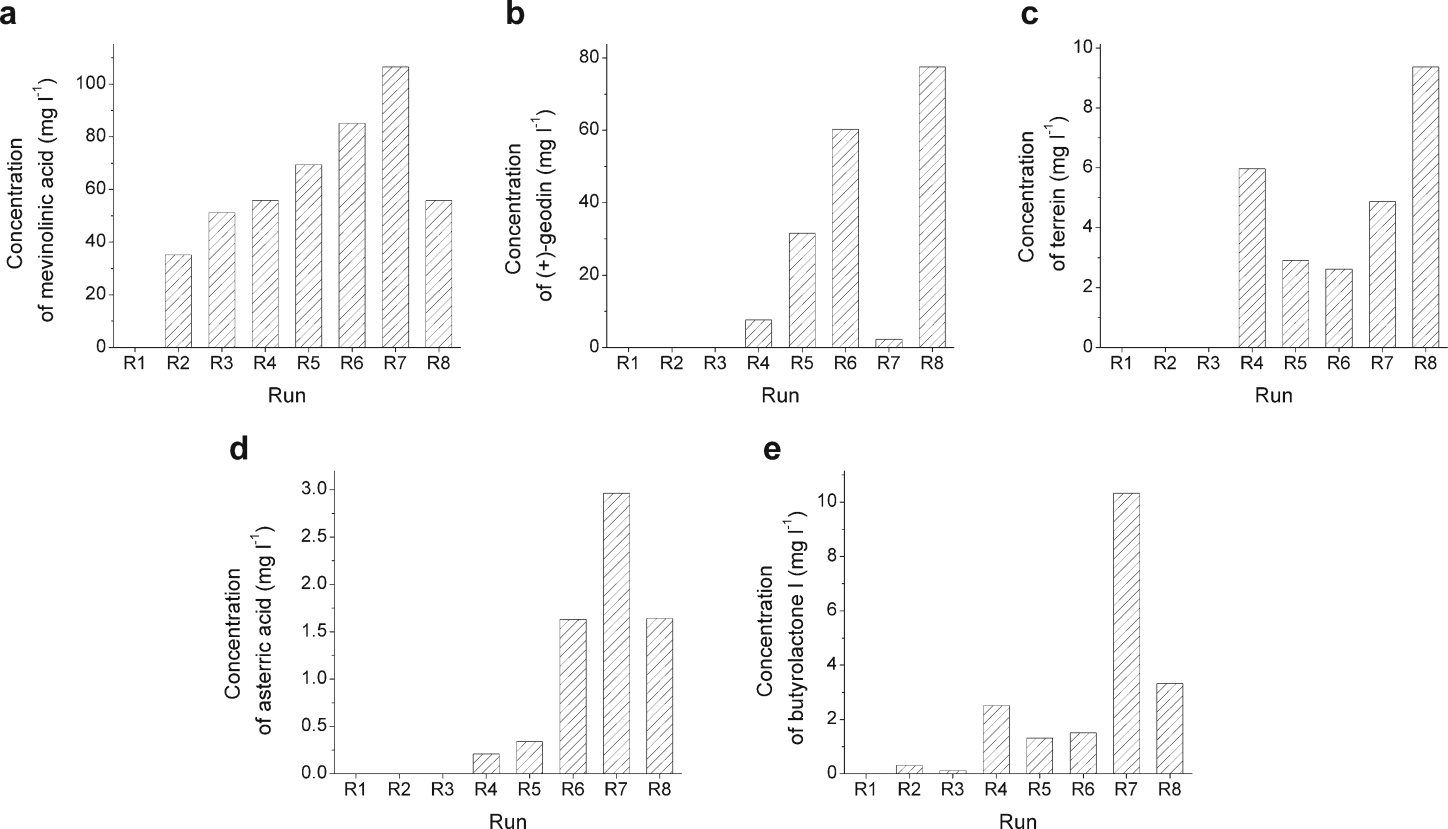
Results of quantitative analysis of mevinolinic acid (**a**), (+)-geodin (**b**), terrein (**c**), asterric acid (**d**), and butyrolactone I (**e**). The highest concentration values observed throughout the duration of each run are reported. The formation of mevinolinic acid, asterric acid, and butyrolactone I was favored in the R7 culture, which involved the supplementation with rapeseed oil and reduction of chlorine availability. The production of (+)-geodin and terrein was enhanced in the R8 culture, which was relatively well aerated during the idiophase. No traces of secondary metabolites were found in the highly saline R1 medium

The production of mevinolinic acid, (+)-geodin, terrein, asterric acid, and butyrolactone I was studied quantitatively by using the calibration curves obtained for standard solutions. The highest observed concentration of each metabolite with relation to the corresponding run is reported in Fig. 3.

The R7 culture, which involved the addition of rapeseed oil with the simultaneous elimination of NaCl from the medium, proved to be most successful in terms of stimulating mevinolinic acid (Fig. 3a), asterric acid (Fig. 3d), and butyrolactone I (Fig. 3e) biosynthesis. While rapeseed oil was also supplemented in the runs R5, R6, and R8, the removal of all chlorine except the amount present in yeast extract was the unique characteristics of R7 culture. The inducing effect of the nutritional strategy tested in R7 was particularly evident in the case of butyrolactone I (Fig. 3e). The concentration of this metabolite reached 10.32 mg l^−1^ in the culture R7, whereas the second highest value, obtained in the run R8, was equal to 3.32 mg l^−1^. Similar observations were made for asterric acid. Its concentration in R7 reached 2.96 mg l^−1^, a value which corresponded to 1.8-fold increase with respect to the values determined for cultures R8 and R6 (Fig. 3d). The R7 culture promoted asterric acid, butyrolacone I, and mevinolinic acid biosynthesis and resulted in the highest observed concentration in the entire study, namely 106.57 mg l^−1^ of mevinolinic acid (Fig. 3a). However, the conditions of the R7 run were clearly unfavorable with respect to the formation of (+)-geodin (Fig. 3b).

**Fig. 4:**
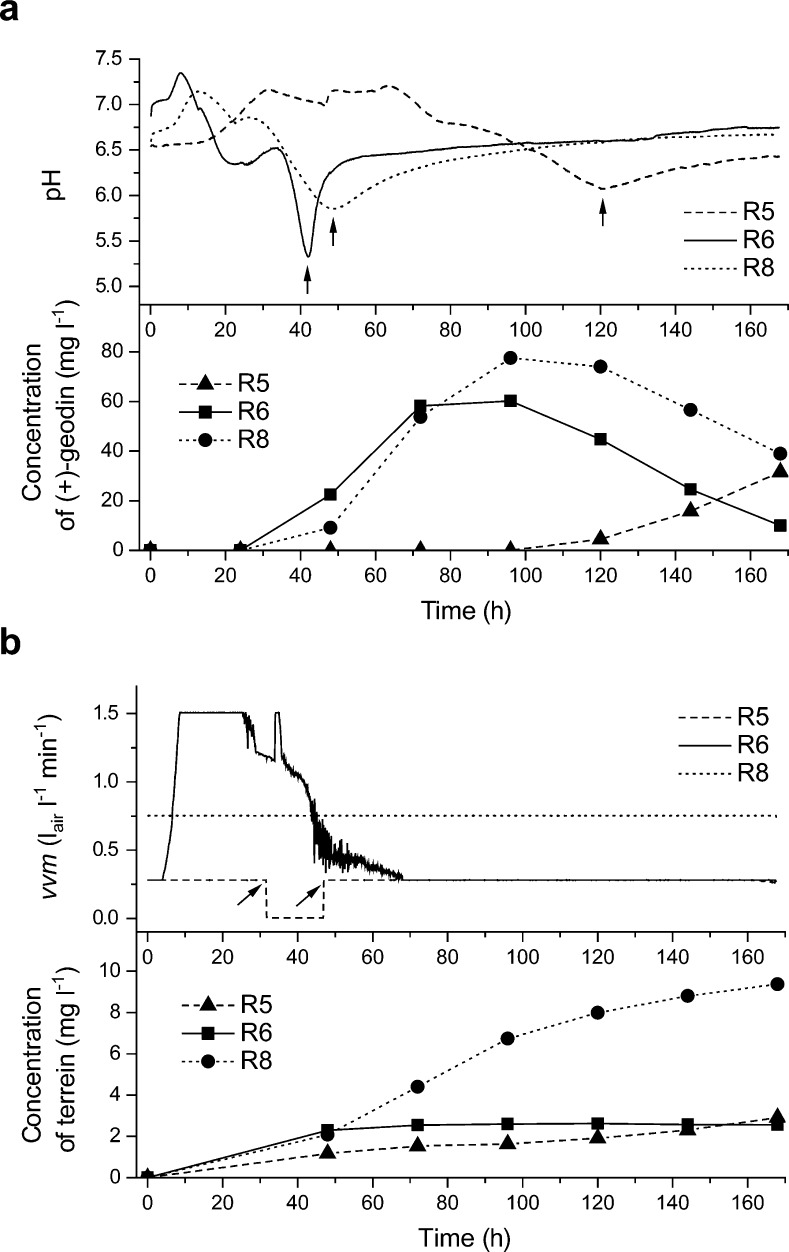
Time courses of (**a**) (+)-geodin biosynthesis with respect to pH of the broth and (**b**) terrein production in terms of the observed *vvm* (ratio of air flow rate and bioreactor volume). **a** The onset of (+)-geodin accumulation roughly corresponds to the inflection of pH observed in the corresponding culture (indicated by arrows). **b** The enhanced production of terrein in the R8 culture correlates with the period of relatively intensive aeration in R8. The 15-h-long shutdown of aeration during the R5 run is indicated by two *arrows*

The R8 culture, which involved the addition of rapeseed oil and constant rate of aeration throughout the run, led to the highest observed concentration of terrein (Fig. 3c) and (+)-geodin (Fig. 3b) equal to 9.37 and 77.53 mg l^−1^, respectively. Even though the medium composition was shared among the cultures R5, R6, and R8, the aeration strategies were different (see Fig. 1). Time series data of R5, R6, and R8 runs was confronted with on-line bioreactor measurements of pH, air flow rate, dissolved oxygen saturation level, impeller speed, and redox potential in order to determine the process-related factors that contributed to inducing terrein and (+)-geodin formation. The following two correlations were observed. Firstly, the onset of (+)-geodin accumulation roughly correlated with the time of pH inflection point (Fig. 4a). This behavior was observed for all three cultures but there was no observable correlation between the magnitude of the pH change and the relative (+)-geodin titers. A different phenomenon was noticed for terrein (Fig. 4b). The concentration of this metabolite in the R8 culture increased steadily throughout the entire run, while no significant production was observed in the R5 and R6 cultures after 48 h of cultivation. Notably, it corresponded to the time when the aeration rate of R6 decreased below the rate of R8. Keeping the aeration rate constant at a relatively high level, from the 48 h until the end of the cultivation, resulted in the increased production of terrein compared to the R5 and R6 cultures (Fig. 4b). All in all, this strategy proved to be more efficient at stimulating biosynthetic routes than shocking the fungus with aeration shutdown and long-term oxygen deficiency in the R5 run, since the concentration of metabolites in the R8 culture turned out to be higher than in oxygen-poor R5 culture, with the single exception of mevinolinic acid (Fig. 3).

The cultures R3 and R4 were characterized by different levels of yeast extract in the medium (Fig. 1). Specifically, the concentration of nitrogen source in the R3 run was eight times higher than in R4. Clearly, under the tested experimental conditions, the limited nitrogen availability promoted the production of mevinolinic acid, (+)-geodin, terrein, asterric acid, and butyrolactone I (Fig. 3). For all five quantified compounds, the metabolite concentration in the nitrogen-deficient R4 culture exceeded the one observed for the nitrogen-richer R3 culture. What is more, the signals corresponding to (+)-geodin, asterric acid, and terrein were not observed at all for the samples originating from the R3 run.

All growth media contained lactose as the main carbon source. The effect of second carbon source addition, namely inulin (R2) and rapeseed oil (R5, R6, R7, and R8), was examined in the context of secondary metabolism induction. The levels of metabolites observed for the oil-containing media were higher than those for the inulin-containing R2 medium (Fig. 3). As in the case of the previously discussed nitrogen-rich medium, no significant signals of (+)-geodin, asterric acid, and terrein were detected for the inulin-containing R2 broth.

**Fig. 5:**
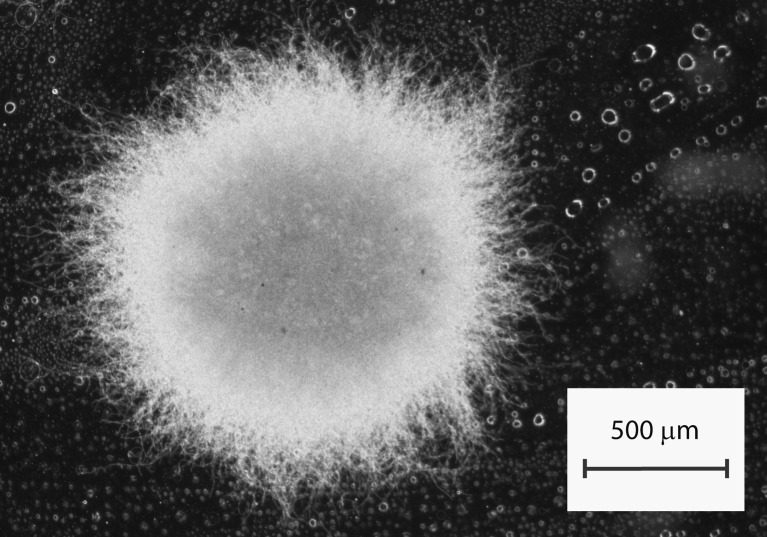
Compact and dense morphology of fungal pellets observed in highly saline R1 culture

The R1 medium containing 15 % of NaCl proved to be strongly inhibitory for secondary metabolism (Fig. 3). No traces of secondary metabolites were detected in this case. Interestingly, the post-inoculation lag phase lasted much longer than for other performed bioreactor runs. The dissolved oxygen saturation level remained close to 100 % until the third day of cultivation, when the growth phase was initiated. Despite the survival of the fungus, the secondary metabolic pathways remained inactive throughout the cultivation period. The formation of exceptionally dense and compact pellets was observed for the R1 culture (Fig. 5).

The remaining metabolites were subjected to qualitative analysis. The presence of 4a,5-dihydromevinolinic acid and 3α-hydroxy-3,5-dihydromonacolin L acid was detected in all examined cultures except the saline environment of R1. Dihydroisoflavipucine was found in all runs except the nitrogen-deficient R4 culture and the salt-rich R1 culture. The production of (+)-erdin, demethylasterric acid, and aspulvinone E was observed in the nitrogen-deficient culture R4 and oil-supplemented cultures R5, R6, R7, and R8. The presence of questin and sulochrin was revealed in the oil-containing media R5, R6, R7, and R8. Finally, the significant signals corresponding to desmethylsulochrin and (+)-bisdechlorogeodin were detected solely under the oil-enriched and chlorine-deficient conditions of R7.

## Discussion

The main outcome of the study was the induction of biosynthesis of 15 secondary metabolites originating from six metabolic pathways of *A. terreus* (Fig. 6). To initiate the flux through the pathways, the corresponding parts of the genome needed to be activated by providing the required stimuli. This was attempted by the diversification of growth media and cultivation conditions. The collection of ESI+ and ESI− spectral data gathered in the course of analytical LC-MS analyses (Table 1; Figs. S1–S15) can be used as a resource in future experiments on *A. terreus* metabolism in the absence of authentic standards.

**Fig. 6:**
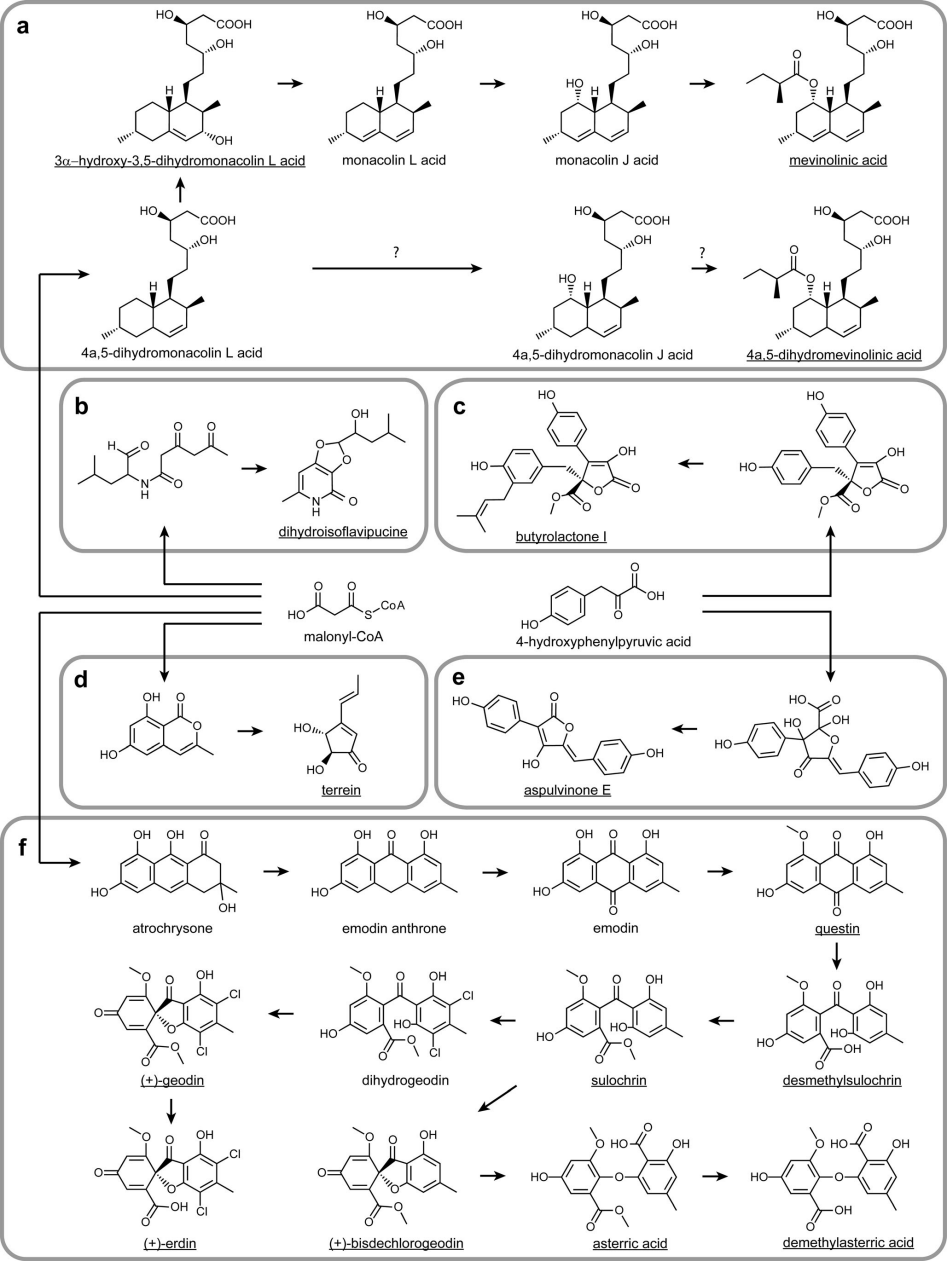
Scheme of biosynthetic origins of secondary metabolites detected in bioreactor cultures of *A. terreus* ATCC 20542 based on the previously published works. The *underlined names* of molecules indicate the metabolites that were encountered in the course of the study. For clarity, some metabolic intermediates are not included in the scheme. Malonyl-CoA is presented as the precursor of polyketide-type compounds (**a**), (**b**), (**d**), and (**f**), while 4-hydroxyphenylpyruvic acid fuels the formation of butyrolactone I (**c**) and aspulvinone E (**e**). **a** 3α-Hydroxy-3,5-dihydromonacolin L acid and mevinolinic acid originate from the common metabolic pathway (Barriuso et al. 2011; Kennedy et al. 1999). It is proposed here that 4a,5-dihydromevinolinic acid may be formed via hydroxylation of 4a,5-dihydromonacolin L acid and subsequent esterification with methylbutyrate (reactions marked by question marks). **f** Eight of out 15 identified molecules have their origin in the common octaketide pathway (Chen et al. 1992; Nielsen et al. 2013). It is suggested here that (+)-erdin (Raistrick and Smith 1936) and demethylasterric acid (Natori and Nishikawa 1962) are the results of hydrolysis of ester bonds in (+)-geodin and asterric acid, respectively. Desmethylsulochrin is a precursor of sulochrin, but its presence can also be attributed to ester bond hydrolysis in sulochrin (not shown). The activity of biosynthetic pathways of (**b**) dihydroisoflavipucine (Gressler et al. 2011), (**c**) butyrolactone I (Guo et al. 2013), (**d**) terrein (Zaehle et al. 2014), and (**e**) aspulvinone E (Guo et al. 2013) have also been observed in the experiment

As expected, mevinolinic acid (Fig. 6a) was the major metabolite identified in the broth, with the relatively high concentration exceeding 100 mg l^−1^. Its formation is a result of coordinated action of two polyketide synthases and a set of post-PKS tailoring enzymes (Barriuso et al. 2011). 3α-Hydroxy-3,5-dihydromonacolin L (Fig. 6a), another molecule detected in the study, was previously shown to originate from the same pathway (Nakamura et al. 1990; Treiber and Reamer 1989). The biosynthetic mechanism of 4a,5-dihydromevinolin formation has not been yet elucidated. It is suggested here that the formation of 4a,5-dihydromevinolin may be a result of single hydroxylation of 4a,5-dihydromonacolin L acid and further esterification with methylbutyrate (Fig. 6a).

The metabolite dihydroisoflavipucine (Fig. 6b) stems from the hybrid polyketide non-ribosomal peptide route involving malonyl-CoA and leucine as precursors (Gressler et al. 2011). The current study is the first report of dihydroisflavipucine formation by the strain *A. terreus* ATCC 20542. Another molecule, butyrolactone I (Fig. 6c), was described in a several strains of *A. terreus* (Nitta et al. 1983; Palonen et al. 2014; Rao et al. 2000), including a reisolate (Schimmel et al. 1998) and mutant (Schimmel and Parsons 1999) of *A. terreus* ATCC 20542. Its formation was proposed previously to originate from 4-hydroxyphenylpyruvic acid and involved the catalytic activity of a non-ribosomal peptide synthetase-like enzyme (Guo et al. 2013). The detection of a polyketide compound derived from 6-hydroxymellein (Zaehle et al. 2014), namely terrein (Fig. 6d), was not surprising, since it was found in *A. terreus* ATCC 20542 agar cultures (Samson et al. 2011) and shake flask cultures (Boruta and Bizukojc 2014). The formation of aspulvinones was mentioned earlier for a number of *A. terreus* strains, including the ATCC 20542 strain cultivated on agar media (Samson et al. 2011). Since aspulvinone E (Fig. 6e) was proposed to be one of the early products of the biosynthetic pathway of aspulvinones (Guo et al. 2013), its presence in the examined bioreactor cultures confirmed the previously reported observations.

Eight metabolites identified in the study, namely questin, desmethylsulochrin, sulochrin, (+)-erdin, (+)-bisdechlorogeodin, (+)-geodin, asterric acid, and demethylasterric acid (Fig. 6f), have their origin in the anthraquinone-related biosynthetic pathway (Chen et al. 1992; Nielsen et al. 2013). It is proposed here that demethylasterric acid (Curtis et al. 1960; Natori and Nishikawa 1962) and (+)-erdin (Raistrick and Smith 1936) result from hydrolysis of ester bonds in asterric acid and (+)-geodin, respectively (Fig. 6f). It is unknown whether the presence of desmethylsulochrin in the broth can be attributed to hydrolysis of sulochrin or rather is a result of direct secretion of desmethylsulochrin, which in fact serves as a precursor for sulochrin in the sequence of biosynthetic steps leading from atrochrysone towards (+)-geodin (Fig. 6f). With regard to attained concentration values, (+)-geodin was confirmed here to be one of the major secondary metabolites produced by *A. terreus* ATCC 20542 (Askenazi et al. 2003; Bizukojc and Ledakowicz 2008). Furthermore, it is the first study that mentions demethylasterric acid as a molecule associated with *A. terreus* ATCC 20542.

The results of the study revealed the significant differences in the biosynthetic repertoire of *A. terreus* ATCC 20542 with regard to the applied bioprocess-related strategies. Notably, the bioreactor runs employing high concentration of NaCl (R1), addition of inulin (R2), and increased supply of yeast extract (R3) were characterized by relatively poor secondary metabolites production. The recent study of Abd Rahim et al. (2015) showed that the addition of 1 g l^−1^ NaCl enhances both lovastatin and (+)-geodin biosynthesis by *A. terreus* ATCC 20542 in glycerol-containing media. In the previous work of Tresner and Hayes (1971) regarding the NaCl tolerance of fungi, the two examined strains of *A. terreus* were shown to tolerate the NaCl levels even above 25 %. The concentration of 150 g l^−1^ (15 % *w*/*v*) NaCl was chosen here to shock the fungal cells in a bioreactor culture. It turned out to be a very demanding environment for *A. terreus* ATCC 20542. The on-line measurements of dissolved oxygen saturation levels were monitored during the two initial days of cultivation, but there was no indication of entering the growth phase. At first, it was not clear whether the investigated culture was capable of adapting to this saline environment. However, osmoadaption did take place as the consumption of oxygen increased after the 48 h of cultivation indicating the start of the growth phase. Apparently, under the tested conditions, the strategy of survival assumed the shutdown of secondary metabolic pathways and investing the available biochemical resources into osmostress-related cellular functions (Duran et al. 2010). This was reflected by the observed morphology (Fig. 5). In comparison to the earlier reports (Bizukojc and Ledakowicz 2010; Gonciarz and Bizukojc 2014), the pellets formed in 15 % NaCl exhibited a remarkably compact and dense structure. This morphology indicated the minimization of contact area between the cells and the medium, what may be crucial for survival in these conditions. On the other hand, it also indicated the reduced transport of oxygen and nutrients into the pellet, what may be associated with the lack of secondary metabolic activity. In fact, this was the only bioreactor culture, for which there were no detected traces of secondary metabolites in the broth. The inhibitory effect of the added inulin and increased amount of nitrogen source was not as strong as for the high salt medium (Fig. 3). The deficiency of nitrogen source was reported previously to have a positive effect on lovastatin and (+)-geodin production by *A. terreus* ATCC 20542 (Bizukojc and Ledakowicz 2007, 2008). Notably, in contrast to other investigated compounds, dihydroisoflavipucine production was not observed in low-nitrogen R4 culture. The current study confirmed the importance of amino acids in the induction of dihydroisoflavipucine production described by Gressler et al. (2011). In the run R4, the deactivation of its biosynthetic pathway occurred due to the insufficient supply of yeast extract, which was a source of amino acids. The relatively high concentration of terrein in the nitrogen-deficient R4 culture confirmed the recent findings of Gressler et al. (2015) that nitrogen starvation induces terrein biosynthesis. However, under the conditions applied here, the stimulatory effect of rapeseed oil in the well-aerated culture R8 led to higher levels of terrein than those in the R4 run, as described below.

The addition of rapeseed oil, which is a mixture of saturated and unsaturated fatty acids, was shown to be an effective strategy to enhance the formation of secondary metabolites in *A. terreus* cultures. Among the quantified metabolites, the highest concentration values were reported either for R7 or R8 runs (Fig. 3), both of which involved the supplementation with oil. Its stimulating effect can be attributed to the increased supply of acetyl-CoA originating from β-oxidation of fatty acids, which in turn fuels the biosynthesis of malonyl-CoA and the downstream molecules (Fig. 6a, b, d, f). Another explanation is that linoleic acid present in rapeseed oil acts as a precursor of oxylipin, which elicits the quorum sensing responses in *A. terreus* and enhances the formation of secondary metabolites, as described by Sorrentino et al. (2010). Abd Rahim et al. (2015) reported previously that the supplementation with oleic acid elevates the concentration of lovastatin and (+)-geodin in *A. terreus* ATCC 20542 cultures cultivated with glycerol as the carbon source. It was also shown before that the addition of linoleic acid enhances lovastatin production by *A. terreus* (Sorrentino et al. 2010). The present study not only confirms the stimulatory effect of fatty acids on biosynthesis of lovastatin and (+)-geodin, but also indicates that the addition of oils can induce biosynthesis of a wide range of secondary metabolites.

The enhancing effect of rapeseed oil on secondary metabolism was investigated in combination with diversified aeration conditions in the bioreactor runs R5, R6, and R8. The R5 culture was maintained in the oxygen-deficient conditions. The shutdown of aeration for 15 h was performed in order to put the cells in the state of shock. According to the results, the oxygen-related stress did not trigger the formation of otherwise absent metabolites, nor did it increase the concentration of quantified molecules. In contrast, the aeration scheme implemented in the R8 run resulted in the highest concentration of (+)-geodin and terrein observed in the study (Fig. 3b, c). Maintaining relatively high and constant aeration rate throughout the idiophase proved to be beneficial for the production of these two metabolites. The correlation between the concentration of (+)-geodin and increased aeration rate was previously reported for *A. terreus* ATCC 20542 by Bizukojc and Ledakowicz (2008). Aeration rate and agitation speed were previously shown to influence the formation of terrein by *A. terreus* strain PF16 (Xu et al. 2012). Keeping the constant air flow and impeller speed turned out to be beneficial for terrein production by the PF16 strain. It agrees with the results obtained in the current study, as there was no oxygen level control in the R8 process and these two values were kept constant (see Fig. 1 and “Materials and methods” section for details). On the other hand, the production of terrein by the PF16 strain was positively influenced by decreasing both the aeration rate and agitation speed (Xu et al. 2012). This behavior was not observed for the ATCC 20542 strain, since air flow rate in the R8 run was higher than that in R5 and R6 cultures when the relatively high level of terrein biosynthesis was observed (Fig. 4b) and the stirrer speed in R8 was kept constant throughout the study at a relatively high level of 240 rpm. With regard to pH measurements in the runs R5, R6, and R8, the pH inflection points corresponding to the onset of (+)-geodin production confirm the observations made previously by Bizukojc and Ledakowicz (2008).

The addition of rapeseed oil was combined with minimization of chlorine content in the R7 culture. The residual amount of Cl^−^ was expected to be present in the supplied yeast extract, but NaCl was present neither in the bioreactor culture nor in the corresponding preculture. This strategy was developed to provoke the reorganization of metabolic fluxes by simultaneously enhancing the activity of secondary pathways and establishing the conditions of chlorine deficiency. The effects of chlorine limitation on the secondary metabolic profile of *A. terreus* have not been reported previously. The limitation of a single element can lead to significant changes in secondary metabolome. For example, the production of patulin by *Penicillium urticae* was previously shown to be dependent on manganese supplementation and the accumulation of the pathway intermediate 6-methylsalicylic acid was reported for the manganese-deficient medium (Scott et al. 1984). In the current study, the limitation of chlorine led to the shutdown of (+)-geodin production, but the trace amounts of this metabolite were detected during the initial 48 h of cultivation. It reflected the fact that the residual amount of chlorine originating from yeast extract was utilized for (+)-geodin biosynthesis in the initial phase of growth and the biosynthetic route was deactivated as soon as the chlorine source was depleted. Similarly, only the weak signals corresponding to (+)-erdin were detected in the samples originating from the R7 culture. The shutdown of (+)-geodin and (+)-erdin formation in the absence of chlorine was anticipated, since these molecules have two chlorine atoms in their structure (Raistrick and Smith 1936). The blocking of (+)-geodin and (+)-erdin formation resulted in the intensified production of butyrolactone I, asterric acid, and mevinolinic acid (Fig. 3). Moreover, it led to the redirection of metabolic fluxes towards the formation of molecules that were not detected in other cultures, namely desmethylsulochrin and (+)-bisdechlorogeodin. The increased production of asterric acid can be explained by the promiscuity of dihydrogeodin oxidase, an enzyme responsible for the transformation of dihydrogeodin into (+)-geodin. Even though dihydrogeodin is a preferred substrate for this enzyme, it is also capable of oxidizing a structurally related compound, namely sulochrin, what leads to the formation of (+)-bisdechlorogeodin, a precursor of asterric acid (Fujii et al. 1987; Huang et al. 1996). In the absence of chlorine, dihydrogeodin was not formed and hence the catalytic activity of dihydrogeodin oxidase was limited to sulochrin oxidation.

For all the five quantified metabolites, their concentrations in the nitrogen-limited R4 culture were higher than those in the nitrogen-rich R3 culture (Fig. 3). It is likely that this effect is mediated by the global transcription factors AreA and AtfA encoded by the genes *areA* and *atfA*, respectively, which were previously shown (Gressler et al. 2015) to be involved in the induction of terrein biosynthesis under nitrogen starvation.

The current study involved several strategies to induce biosynthesis of secondary metabolites by *Aspergillus* sp., some of which were not suggested before. This is the first report of testing oil supplementation under diverse aeration schemes and combining the inducing effect of oil with the single element (chlorine) deficiency to trigger biosynthetic pathways. The current study confirms that the previously suggested strategies based on the supplementation with fatty acids (Abd Rahim et al. 2015; Sorrentino et al. 2010) and the cultivation under nitrogen-limited conditions (Scherlach et al. 2011) are useful to uncover the secondary metabolic repertoire of filamentous fungi. While the diversification of carbon sources (Bode et al. 2002; Frisvad 2012) and the cultivation at high salt concentration (Wang et al. 2011) are established methods of inducing biosynthetic pathways, the addition of inulin and providing osmotic stress turned out to be ineffective under the conditions applied here.

In addition to suggesting general strategies to induce secondary metabolism in filamentous fungi, the study provided insights into the selective production of chosen metabolites. For instance, the results show that the oil supplementation under chlorine deficiency is a method to redirect the flux towards the production of asterric acid.

Even though the metabolites presented here, with the exception of lovastatin, are not the subjects of large-scale industrial production, their potential medical relevance remains an open question. The biological activity of these molecules is still a subject of research (Lee et al. 2002; Ohashi et al. 1992; Zaehle et al. 2014; Zhang et al. 2015). While the goal of the study was to induce biosynthesis of metabolites, the presented results are also relevant in the context of elimination of by-products during lovastatin production.

In conclusion, *A. terreus* ATCC 20542 is capable of producing several structurally varied secondary metabolites in a stirred tank bioreactor. The activation of their biosynthesis is highly dependent on the medium composition and the applied cultivation strategy. Providing the stimuli associated with fatty acid supplementation, chlorine deficiency, and optimal aeration is an effective approach for the induction of secondary metabolites biosynthesis by the investigated strain. The culture of *A. terreus* ATCC 20542 remains viable in the liquid medium containing 15 % of NaCl, but the secondary metabolic pathways of the fungus are inoperative under these conditions.

## Electronic supplementary material

ESM 1 (PDF 481 kb)
