# Supplementary material for: Induction of secondary metabolism of Aspergillus terreus ATCC 20542 in the batch bioreactor cultures
Source: Appl Microbiol Biotechnol. 2015 Nov 25;100:3009–22. doi: 10.1007/s00253-015-7157-1 (PMC4786612; doi:10.1007/s00253-015-7157-1)
Supplement: Supplementary file 1 — (PDF 481 kb) [file 253_2015_7157_MOESM1_ESM.pdf]

Supplementary material

Applied Microbiology and Biotechnology

"Induction of secondary metabolism of *Aspergillus terreus* ATCC 20542 in the batch bioreactor cultures"

Tomasz Boruta[*], Marcin Bizukojc

Lodz University of Technology

Faculty of Process and Environmental Engineering

Department of Bioprocess Engineering

ul. Wolczanska 213, 90-924 Lodz, Poland

[*] Corresponding author. Phone: +48 42 631 39 77; fax: +48 42 636 56 63.

E-mail address: tomaszboruta85@gmail.com

Table of contents

Fig. S1 ESI+ (a) and ESI- (b) mass spectra of (+)-bisdechlorogeodin 3

Fig. S2 ESI+ (a) and ESI- (b) mass spectra of (+)-erdin 3

Fig. S3 ESI+ (a) and ESI- (b) mass spectra of (+)-geodin 3

Fig. S4 ESI+ (a) and ESI- (b) mass spectra of 3α-hydroxy-3,5-dihydromonacolin L acid 4

Fig. S5 ESI- mass spectrum of aspulvinone E 4

Fig. S6 ESI+ (a) and ESI- (b) mass spectra of asterric acid 4

Fig. S7 ESI- mass spectra of butyrolactone I 5

Fig. S8 ESI+ (a) and ESI- (b) mass spectra of demethylasterric acid 5

Fig. S9 ESI+ (a) and ESI- (b) mass spectra of desmethylsulochrin 5

Fig. S10 ESI+ mass spectrum of dihydroisoflavipucine 6

Fig. S11 ESI+ (a) and ESI- (b) mass spectra of 4a,5-dihydromevinolinic acid 6

Fig. S12 ESI+ (a) and ESI- (b) mass spectra of mevinolinic acid 6

Fig. S13 ESI+ (a) and ESI- (b) mass spectra of questin 7

Fig. S14 Mass spectra of sulochrin 7

Fig. S15 ESI+ mass spectra of terrein 8

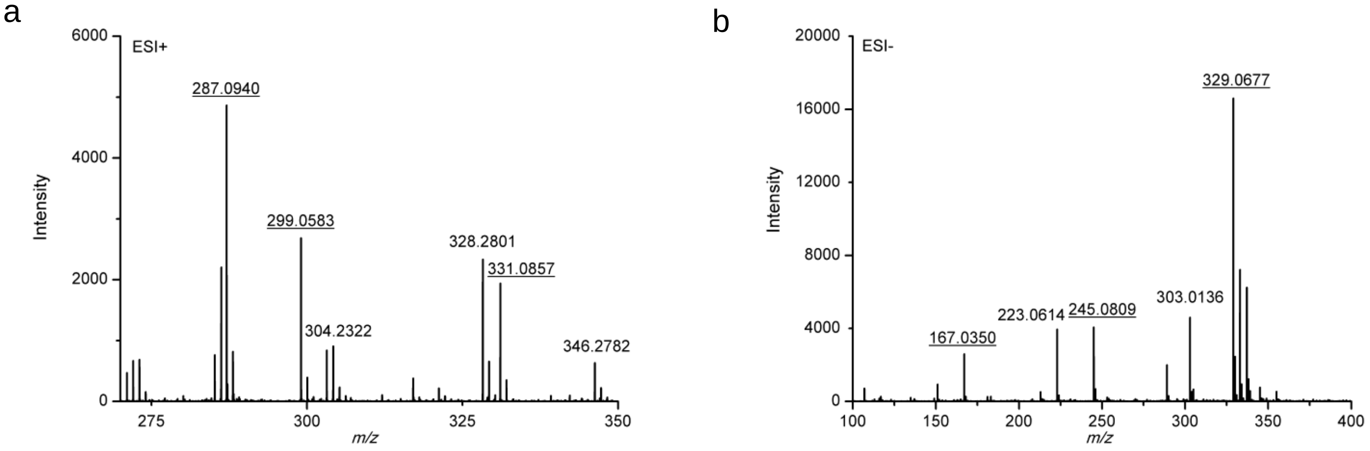

Fig. S1 ESI+ (a) and ESI- (b) mass spectra of (+)-bisdechlorogeodin. The *m/z* values of the peaks corresponding to (+)-bisdechlorogeodin are underlined

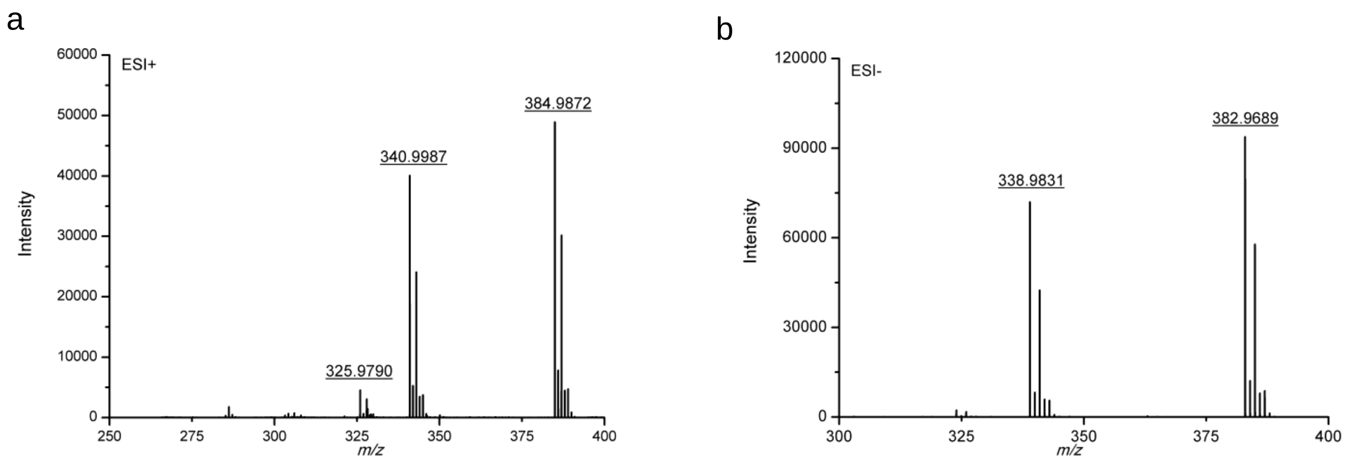

Fig. S2 ESI+ (a) and ESI- (b) mass spectra of (+)-erdin. The *m/z* values of the peaks corresponding to (+)-erdin are underlined

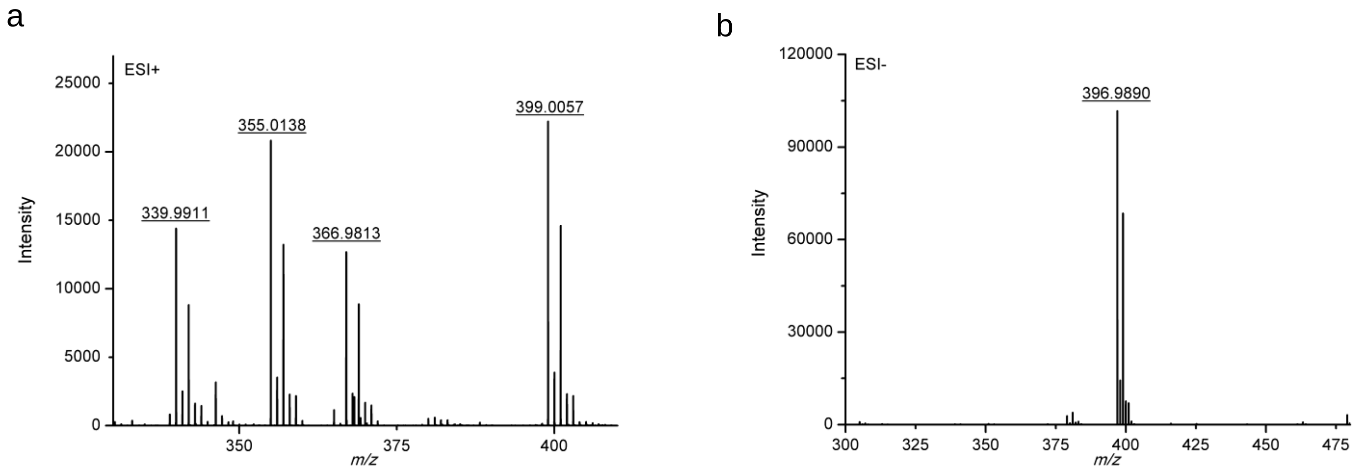

Fig. S3 ESI+ (a) and ESI- (b) mass spectra of (+)-geodin. The *m/z* values of the peaks corresponding to (+)-geodin are underlined

a

b

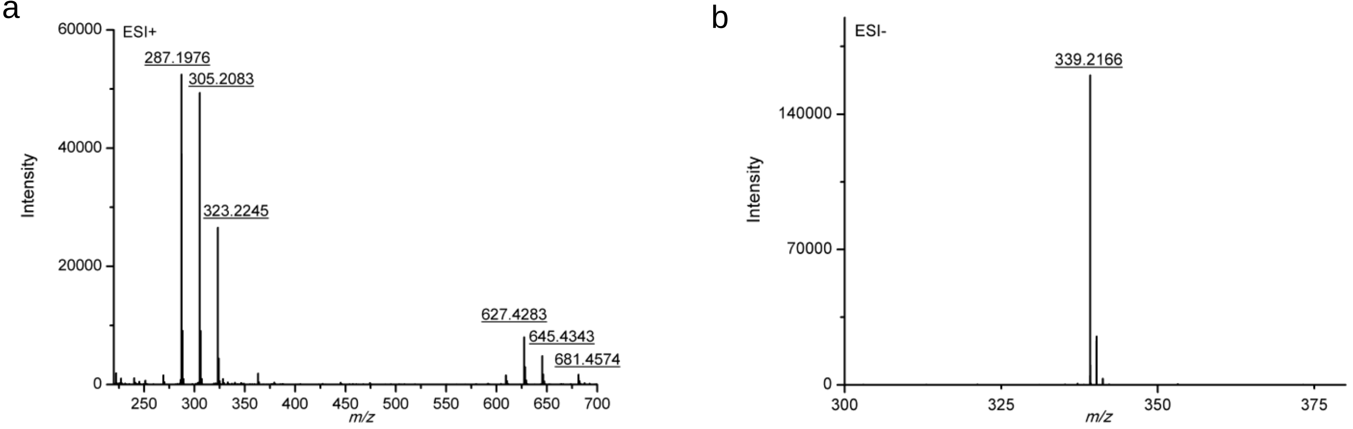

Fig. S4 ESI+ (a) and ESI- (b) mass spectra of 3α-hydroxy-3,5-dihydromonacolin L acid. The *m/z* values of the peaks corresponding to 3α-hydroxy-3,5-dihydromonacolin L acid are underlined

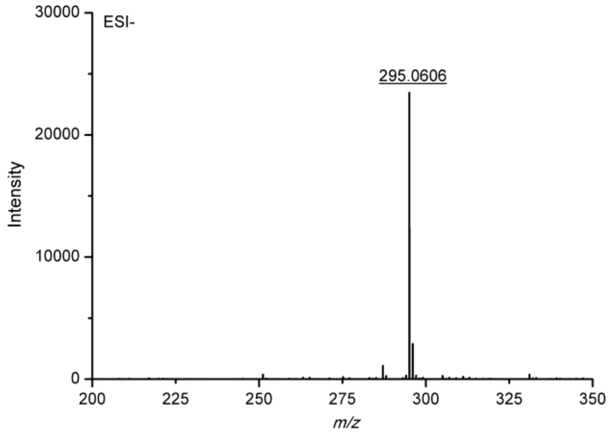

Fig. S5 ESI- mass spectrum of aspulvinone E. The *m/z* value of the peak corresponding to aspulvinone E is underlined

a

b

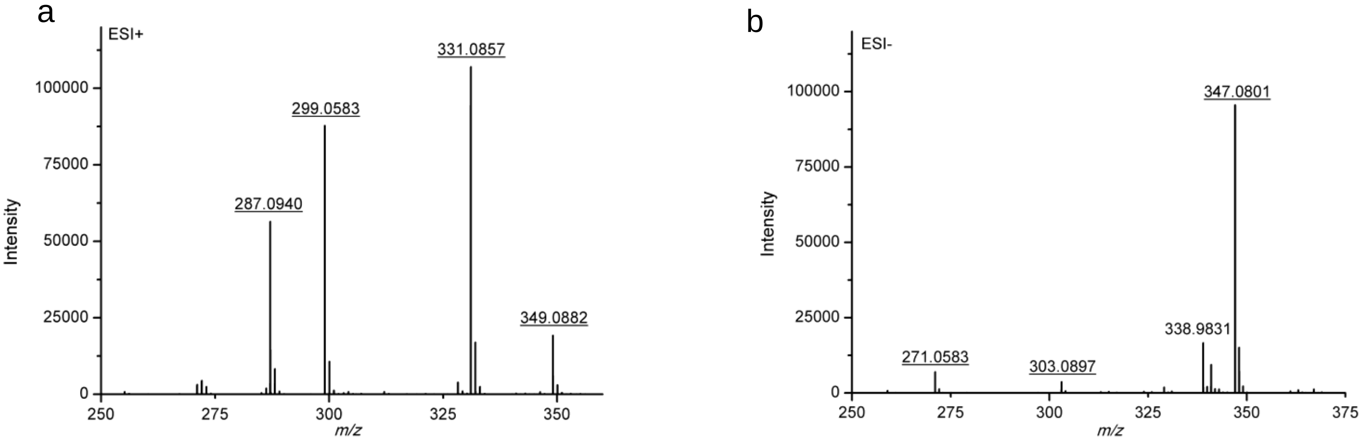

Fig. S6 ESI+ (a) and ESI- (b) mass spectra of asterric acid. The *m/z* values of the peaks corresponding to asterric acid are underlined

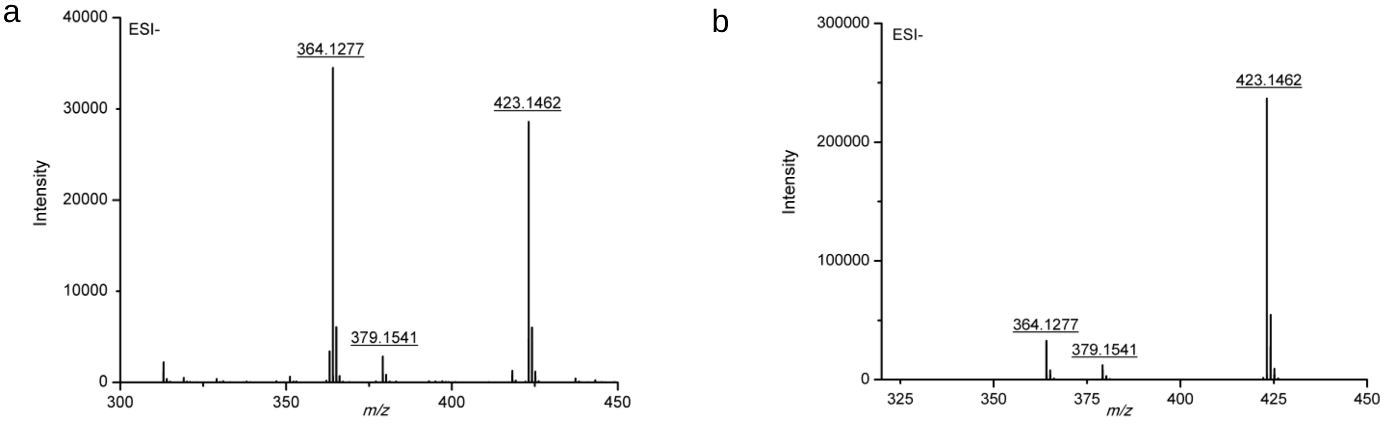

Fig. S7 ESI- mass spectra of butyrolactone I. The *m/z* values of the peaks corresponding to butyrolactone I are underlined. (a) mass spectrum obtained for the analyzed sample; (b) mass spectrum obtained for the standard solution

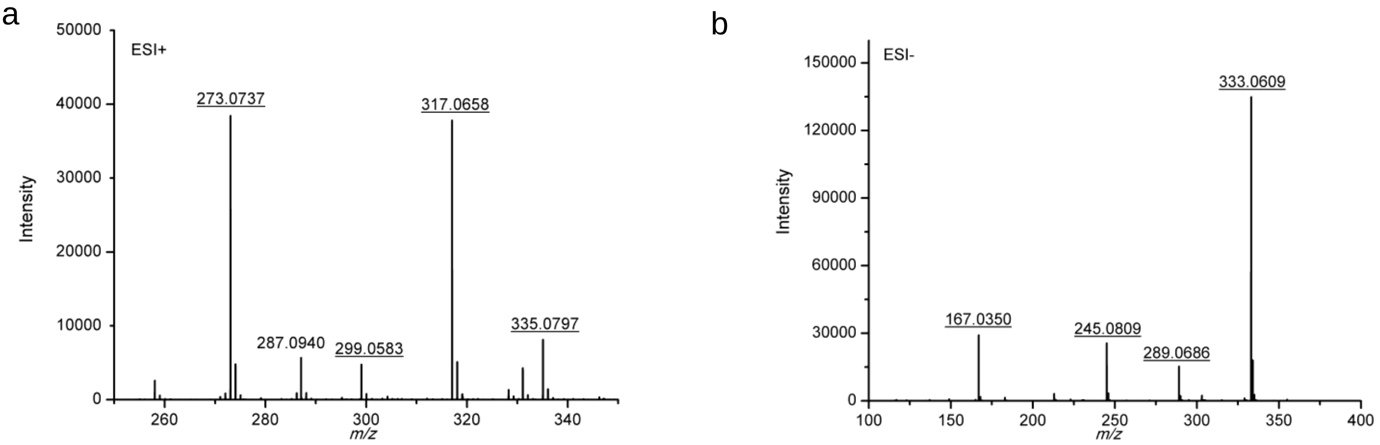

Fig. S8 ESI+ (a) and ESI- (b) mass spectra of demethylasterric acid. The *m/z* values of the peaks corresponding to demethylasterric acid are underlined

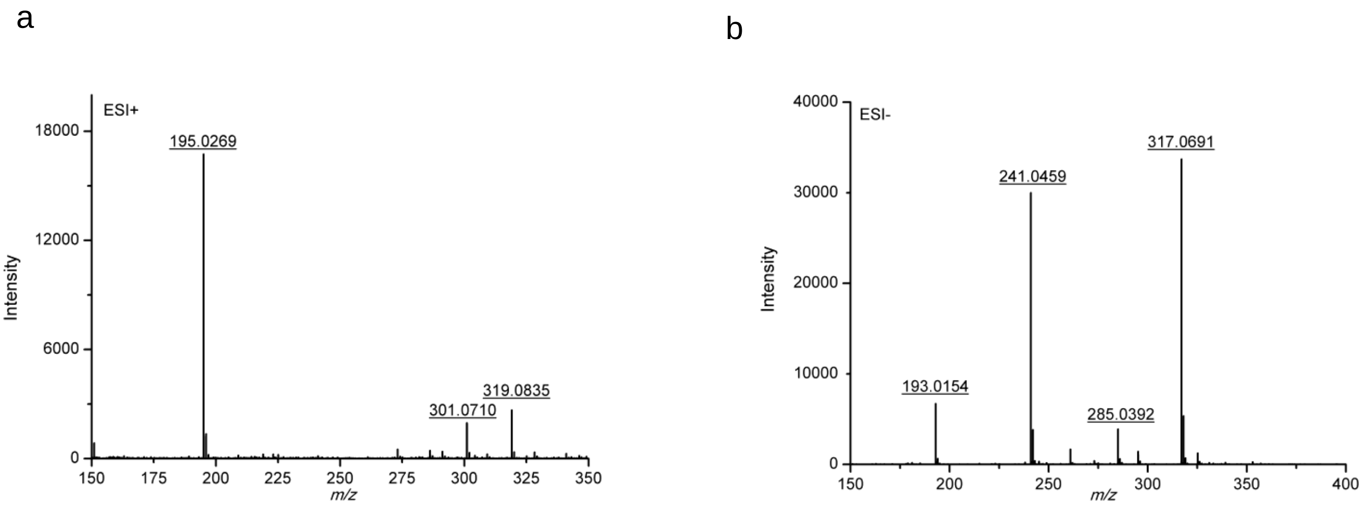

Fig. S9 ESI+ (a) and ESI- (b) mass spectra of desmethylsulochrin. The *m/z* values of the peaks corresponding to desmethylsulochrin are underlined

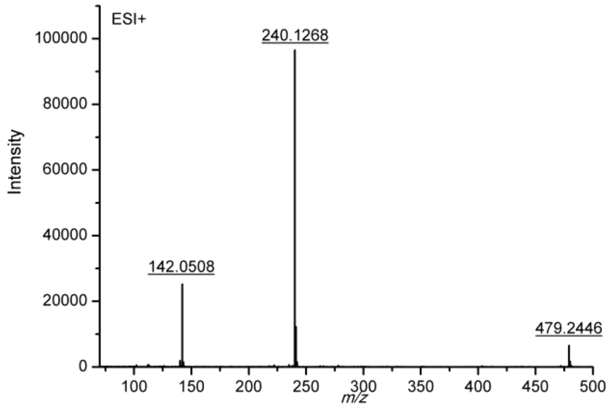

Fig. S10 ESI+ mass spectrum of dihydroisoflavipucine. The *m/z* values of the peaks corresponding to dihydroisoflavipucine are underlined

a b

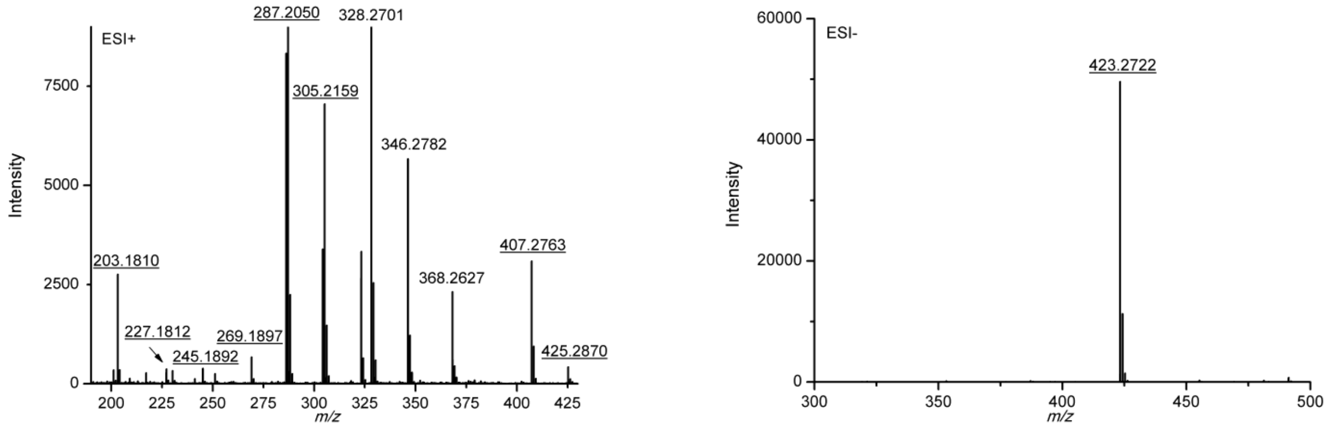

Fig. S11 ESI+ (a) and ESI- (b) mass spectra of 4a,5-dihydromevinolinic acid. The *m/z* values of the peaks corresponding to 4a,5-dihydromevinolinic acid are underlined

a b

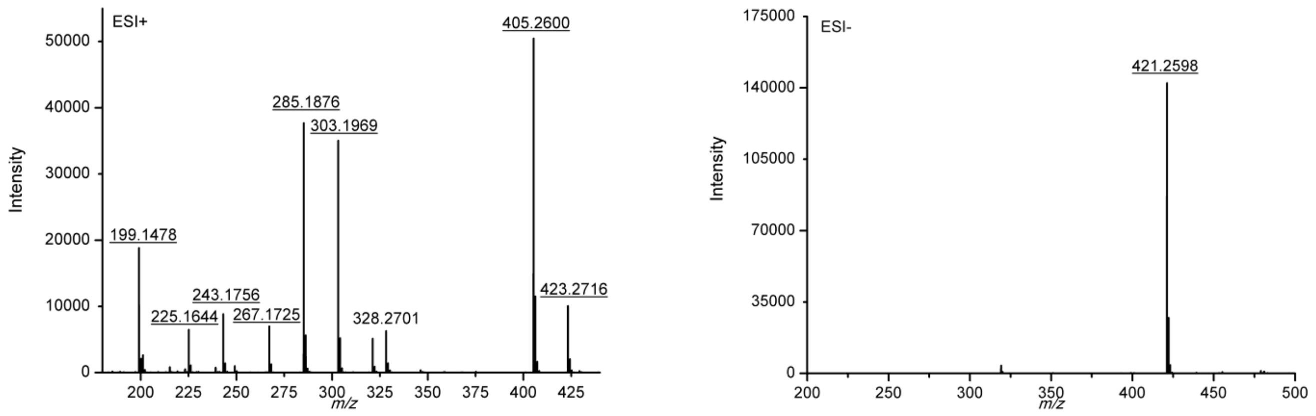

Fig. S12 ESI+ (a) and ESI- (b) mass spectra of mevinolinic acid. The *m/z* values of the peaks corresponding to mevinolinic acid are underlined

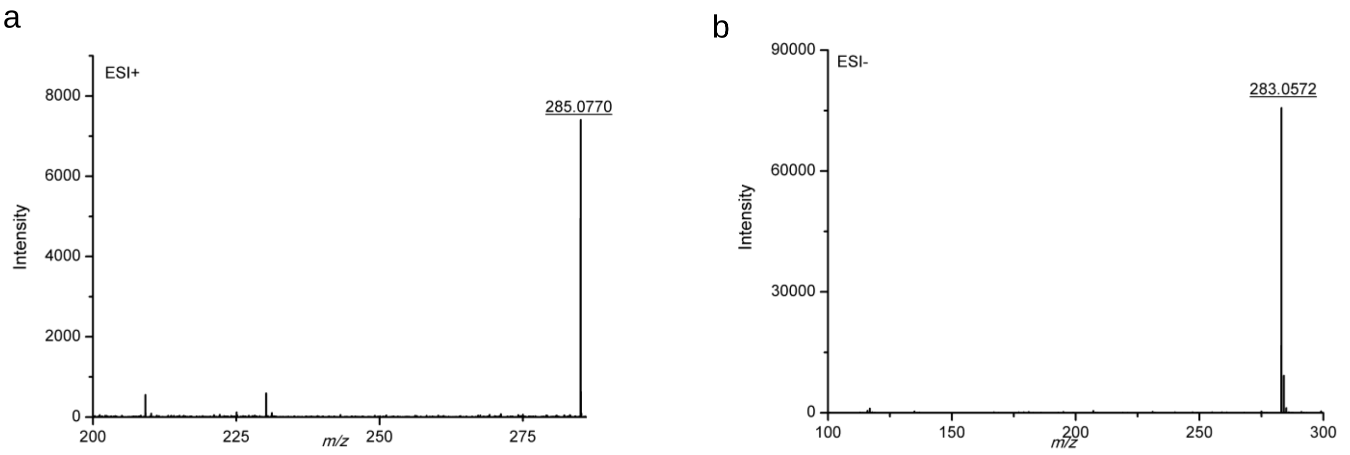

Fig. S13 ESI+ (a) and ESI- (b) mass spectra of questin. The *m/z* values of the peaks corresponding to questin are underlined

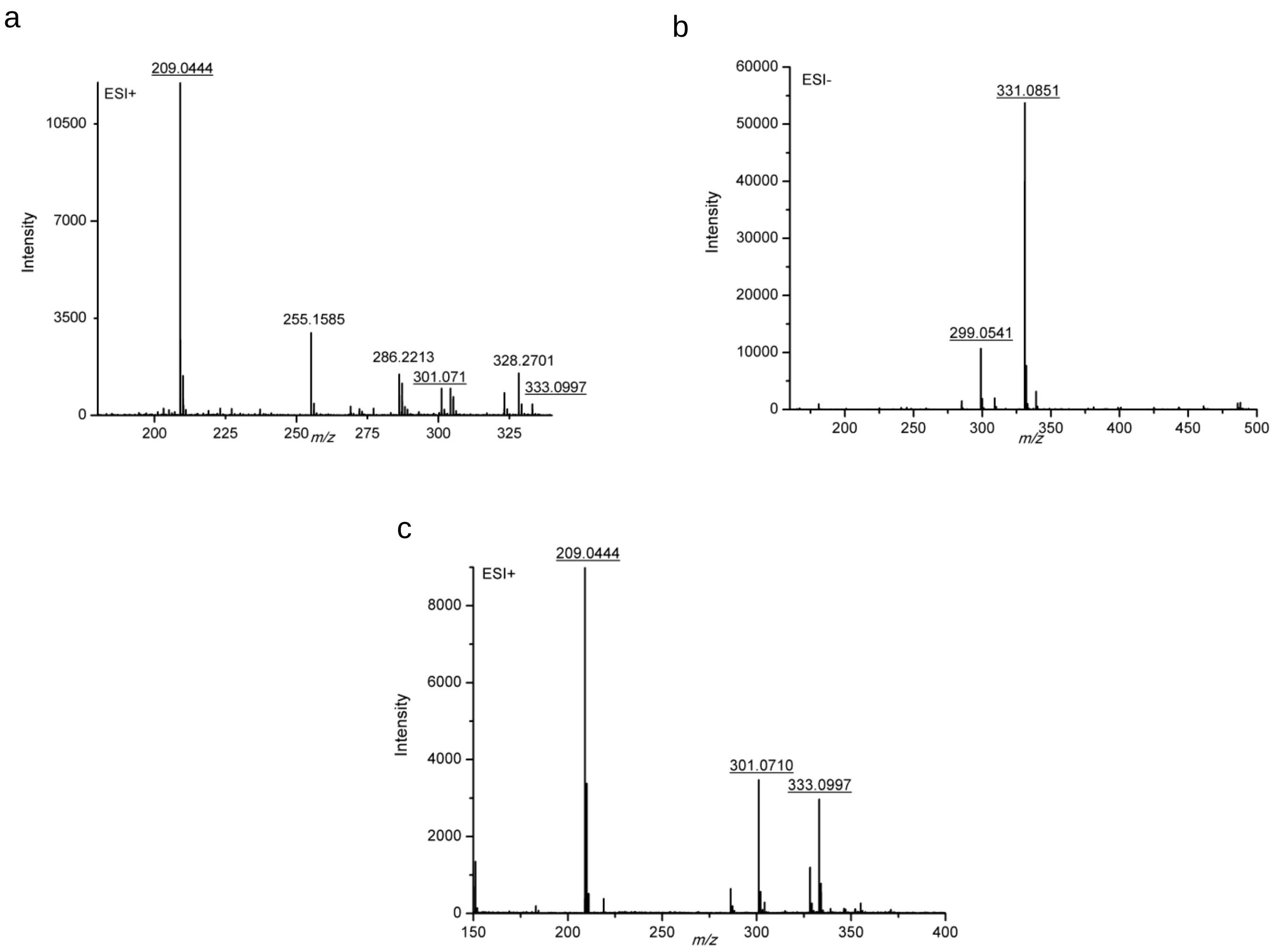

Fig. S14 Mass spectra of sulochrin. The *m/z* values of the peaks corresponding to sulochrin are underlined. (a) ESI+ mass spectrum obtained for the analyzed sample; (b) ESI- mass spectrum obtained for the analyzed sample (c) ESI+ mass spectrum obtained for the standard solution

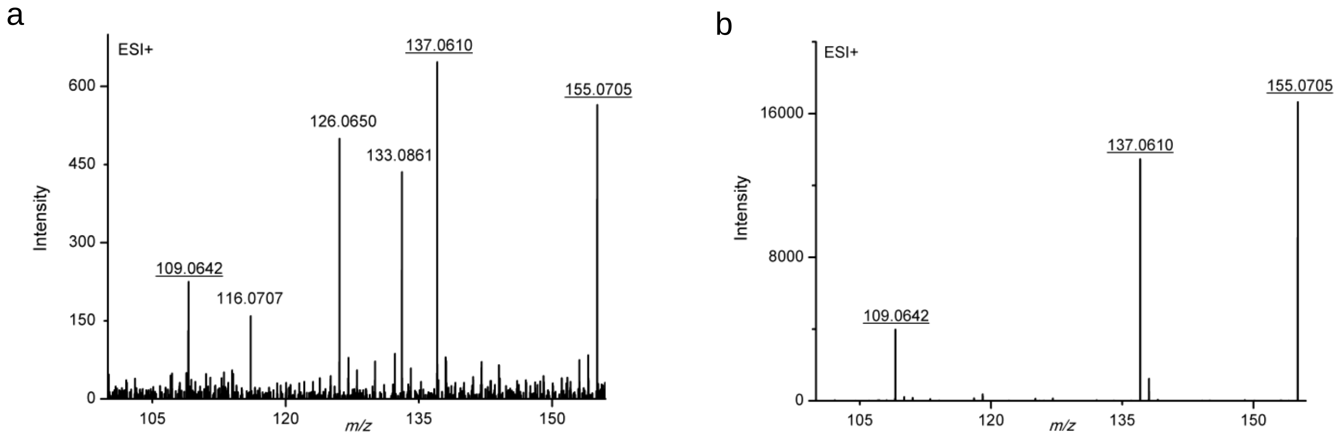

Fig. S15 ESI+ mass spectra of terrein. The *m/z* values of the peaks corresponding to terrein are underlined. (a) mass spectrum obtained for the analyzed sample; (b) mass spectrum obtained for the standard solution
